# HAMP predicts a pivotal role in modulating the malignant behaviors of non-small cell lung cancer cells

**DOI:** 10.18632/aging.205819

**Published:** 2024-05-22

**Authors:** Zhifeng Li, Jinglei Liu, Ping Wang, Boyu Zhang, Guanghui He, Liwei Yang

**Affiliations:** 1 Department of Thoracic Surgery, The Fourth Hospital of Hebei Medical University, Shijiazhuang 050000, China; 2 Department of Respiratory Medicine, The Fourth Hospital of Hebei Medical University, Shijiazhuang 050000, China

**Keywords:** HAMP, proliferation, non-small cell lung cancer, lncRNA, miR-873-5p

## Abstract

Background: Hepcidin antimicrobial peptide (HAMP) is a small peptide hormone recognized for its role in iron metabolism and cancer treatment. The purpose of this study was to examine the influence of HAMP in NSCLC.

Methods: The profile of NSCLC cells and tissues was characterized via HAMP. Gain- or loss-of-function cell models of HAMP were constructed, and CCK8, colony formation, and Transwell analyses were used to confirm the influence of HAMP on NSCLC cells. Upstream and downstream HAMP mechanisms in NSCLC were also analysed. Dual-luciferase reporter and pull-down assays confirmed the associations of miR-873-5p with HAMP, miR-873-5p, and the lncRNA KCNQ1OT1/SNHG14/XIST. Moreover, a xenograft model was established in nude mice for confirming the role of HAMP in NSCLC cell growth.

Results: In addition, HAMP expression increased in NSCLC cells and tissues. In terms of cellular functions, the HAMP-overexpressing group exhibited elevated NSCLC cell proliferation, invasion, and migration. HAMP knockdown reversed these changes. Bioinformatics analysis indicated that miR-873-5p targeted HAMP, which affected the nuclear factor kappa B (NF-κB) pathway in NSCLC. HAMP activated the NF-κB pathway, which was negatively modulated by miR-873-5p. NF-κB inhibitor JSH-23 can partly suppress the proliferation, invasion, and migration in HAMP-overexpressed cells. Moreover, miR-873-5p was the target miRNA of long noncoding RNAs (lncRNAs), which included KCNQ1OT1, SNHG14, and XIST, and these three lncRNAs promoted HAMP.

Conclusion: Noncoding RNA-mediated HAMP promotes NSCLC cell proliferation, migration, and invasion by initiating the NF-κB pathway.

## INTRODUCTION

Non-small cell lung cancer (NSCLC), the predominant form of lung cancer, constitutes approximately 80% of cases, yet the five-year survival rate is only 15% [1, 2]. NSCLC treatment has developed during the last 30 years; however, there are significant challenges, including tumor heterogeneity, drug resistance, and limited treatment options [3, 4]. While targeted therapies, such as EGFR inhibitors and ALK inhibitors, have shown effectiveness in treating subsets of NSCLC patients with specific genetic alterations, the majority of NSCLC patients do not have actionable mutations. This limitation necessitates the exploration of alternative therapeutic strategies and the development of novel treatment options [5, 6]. Hence, determining the molecular intricacies of lung cancer treatment holds substantial significance in the quest for novel pharmaceuticals and enhancing patient survival rates.

Hepcidin antimicrobial peptide, also known as HAMP, is a small peptide hormone that is primarily recognized for its role in iron metabolism and infectious disease defense [7]. Emerging indications allude to the potential involvement of HAMP in the genesis and progression of cancer. For instance, HAMP has been described as having tumor suppressor activity due to its ability to inhibit cell proliferation and growth [8]. HAMP has been implicated in cancer metastasis. Its expression is increased in renal cell carcinoma, as indicated by multiple RNA-seq and cDNA microarray dataset analyses. Elevated HAMP levels were strongly correlated with promoter hypomethylation and immune checkpoint factor expression, suggesting unfavorable survival outcomes among individuals with renal cancer. Following interleukin-34 (IL34) treatment, hepcidin expression is strongly promoted in renal cancer cells. Patients demonstrating augmented HAMP expression achieved this outcome [9]. Dysregulation of the HAMP profile is correlated with altered immune cell function and might promote an immunosuppressive environment that facilitates tumor growth and evasion of immune surveillance [10]. Hence, HAMPs might affect the progression of NSLCC.

Long noncoding RNAs (lncRNAs) are extended noncoding RNAs that surpass 200 nucleotides and are devoid of protein-coding capability [11]. An accumulating body of data hints at the potential of lncRNAs to function as modulators in nearly every cellular process and are intricately intertwined with physiological shifts within organisms and a spectrum of disorders, encompassing malignancies [12–14]. The study of lung cancer-related lncRNAs is still an emerging field, and only a few lncRNAs, such as the lncRNA MALAT1 [15], the lncRNA UCA1 [16], the lncRNA HNF1A-AS1 [17], and the lncRNA PVT1 [18], have been proven to exert biological function and clinical significance. Understanding the functional roles and operational mechanisms of these lncRNAs within the context of NSCLC progression may provide insight into pioneering therapeutic approaches and the detection of plausible diagnostic and prognostic indicators.

Abbreviated noncoding RNA entities modulate gene expression by binding to messenger RNAs (mRNAs), either hindering their translation or fostering their degradation. Aberrant expression or dysregulation of miRNAs has been observed in NSCLC [19, 20]. miRNAs and lncRNAs can interact and modulate each other’s functions in a regulatory network. miRNAs can target lncRNAs, leading to their degradation or repression, while lncRNAs can function as “sponges” or competitive endogenous RNAs (ceRNAs) to sequester miRNAs, thereby modulating the availability and function of miRNAs. This interplay between miRNAs and lncRNAs creates complex regulatory mechanisms that can influence cancer-related gene expression programs [21, 22].

In the present study, we found that HAMP expression was elevated in NSCLC tissues. Based on bioinformatics analysis, miR-873-5p was confirmed to be an upstream target of HAMP, and the lncRNAs XIST, KCNQ1OT1, and SNHG14 potentially target miR-873-5p. Interestingly, these three lncRNAs can promote HAMP mRNA and protein expression. The core objective of this study was to comprehensively scrutinize the repercussions of dampening the KCNQ1OT1/SNHG14/XIST-miR-873-5p-HAMP axis on NSCLC cell proliferation, infiltration, and motility. Additionally, this investigation explored its potential as a novel avenue for forthcoming NSCLC treatment.

## MATERIALS AND METHODS

### Collection of clinical specimens

In this study, we recruited 45 individuals diagnosed with NSCLC, with an average age of 54.15 ± 12.63 years, from the Fourth Hospital of Hebei Medical University. Tumor specimens and adjacent noncancerous tissues were cryopreserved in liquid nitrogen. The Ethics Committee of the Fourth Hospital of Hebei Medical University granted approval for all the samples employed in the experiments (Approval No. FHHM-EC-2023-0021), and patient consent was duly obtained through signed documents.

### Cell culture and transfection

The lung cancer cell lines NCI-H292, NCI-H460, PC9, A549, and NCI-H1975; the normal pulmonary bronchial epithelial cell line BEAS2B; and 293T cells in liquid nitrogen were subjected to a water temperature range of 37°C to 42°C. The cells were cultured in a medium of RPMI-1640 or DMEM-F12 (Solarbio, Beijing, China), which was added with 10% fetal bovine serum (Cat. No. 10270106, Gibco) and 1% penicillin and streptomycin (Solarbio, Beijing, China). The cell culture plates or dishes were put in an incubator with 5% CO_2_ at 37°C. These cells were stirred, lysed, and then subjected to additional centrifugation at 1200 rpm for two minutes at room temperature (RT). Following supernatant removal, the cells were cultivated in 10% serum and double antibody and then blown with the tip of a gun (1 ml) to form a single-cell suspension. Subsequently, the cells were distributed into a plate with an ample supply of fresh culture medium and incubated in a controlled, moisture-rich chamber at 37°C with 5% carbon dioxide. At 24 h prior to transfection, cells (in good growth status) were seeded into 6-well plates at 5 × 10^6^ cells/well. Upon achieving cellular confluence within the range of 70% to 90%, the transfection procedure was executed. The plasmids of pcDNA-HAMP, pcDNA-KCNQ1OT1, pcDNA-SNHG14, pcDNA-XIST, short hairpin RNA targeting HAMP (sh-HAMP), miR-873-5p mimics, and their corresponding negative controls (NCs, including vector, sh-NC, miR-NC) were designed and synthesized by GenePharma (Shanghai, China) Lipofectamine™ 2000 (Cat. No. 11668030, Thermo Fisher Scientific, Inc., Waltham, MA, USA) reagent protocol. Following 48 hours of transfections, the culture medium was removed and exchanged by new fresh medium. After another 24 hours of culture, qPCR or western blot was performed to test HAMP, KCNQ1OT1, SNHG14, XIST or miR-873-5p for confirming the transfection efficiency. The NF-κB inhibitor JSH-23 (10 μM, Cat. No. HY-13982, MedChemExpress, Monmouth Junction, NJ, USA) was used for inhibiting NF-κB activation.

### Real-time qPCR

Total RNA was extracted from cell lines or tissues with TRIzol (Cat. No. 15596026, Invitrogen; Thermo Fisher Scientific, Inc., Waltham, MA, USA). The MolPure^®^ Cell/Tissue miRNA Kit (Cat. No. 19331ES08, Yeasen, Shanghai, China) was used for isolate miRNA from the cells and tissues. For the determination of RNA content, NanoDrop™ One UV spectrophotometry (Thermo Fisher Scientific, Inc.) was used. The extracted RNA was utilized for the synthesis of cDNA through the PrimeScript™ transcription kit (Cat. No. RR036A, Takara Biotechnology Co., Ltd., Japan) or Hifair^®^ miRNA 1st Strand cDNA Synthesis Kit (Cat. No. 11148ES10, Yeasen, Shanghai, China). The procedures were conducted under the instructions of the producer. SYBR Green PCR Master Mix (Cat. No. S7563, Thermo Fisher Scientific, Inc., Waltham, MA, USA) or Hieff^®^ miRNA Universal qPCR SYBR Master Mix (Cat. No. 11170ES03, Yeasen, Shanghai, China) was used for conducting PCR. The thermocycling conditions included: 10 min for reaction at 95°C; 40 cycles of 95°C for 15 sec; 60°C for 45 sec. The relative expression of genes is presented as 2^−ΔΔCT^ method. U6 was used as the internal control of miR-873-5p. GAPDH was used as the internal control of the other detected genes. Each sample was evaluated in triplicate. The PCR primer sequences were listed in Table 1.

**Table 1:** PCR primer sequences.

**Gene**	**Primer sequence**
miR-873-5p	F:5′-GCAGGAACTTGTGAG-3′
	R:5′-GTGCAGGGTCCGAGGT-3′
U6	F:5′-CTCGCTTCGGCAGCACA-3′
	R:5'-AACGCTTCACGAATTTGCGT-3′
KCNQ1OT1	F:5′-CTTTGCAGCAACCTCCTTGT-3′
	R:5′-TGGGGTGAGGGATCTGAA-3′
SNHG14	F:5′-TGAGCGGCATAGATGGTGAC-3′
	F:5′-AGCTCAGTGCAGCAGACCAG-3′
XIST	F:5′-CAGACGTGTGCTCTTC-3′
	R:5′-CGATCTGTAAGTCCACCA-3′
HAMP	F:5′-TCAGTCCCTGTTTGTGAGTCT-3′
	R:5′-AAGGTGGCCCCAATGTTTCC-3′
GAPDH	F:5′-TGTTCGTCATGGGTGTGAAC-3′
	R:5′-ATGGCATGGACTGTGGTCAT-3′

### Dual-luciferase reporter gene system

Plasmids for both the wild-type and mutant variants of KCNQ1OT1/SNHG14/XIST or HAMP were meticulously assembled. The KCNQ1OT1-WT/KCNQ1OT1-Mut, SNHG14-WT/SNHG14-Mut, XIST-WT/XIST-Mut or HAMP-WT/HAMP-Mut recombinant plasmids (GenePharma, Shanghai, China) were extracted by and transfected together with miR-873-5p mimics into 293T cells with the help of Lipofectamine™ 2000 (Cat. No. 11668030, Thermo Fisher Scientific, Inc., Waltham, MA, USA). After a 48-hour interval, the cell culture medium was removed, and the cells were thoroughly rinsed with PBS. Subsequently, 20 μl of cell lysate was added to initiate cell lysis, after which luciferase activity was assessed using a Dual-Luciferase reporter assay system (Promega, Madison, WI, USA).

### Cell counting Kit-8 (CCK-8) assay

H460 and PC9 cells were seeded in 96-well plates at a concentration of 1 × 10^4^ cells per well, with each well containing 200 μL of cell suspension. The cells were allowed to incubate for a period of 24 hours. Next, 10 μL of CCK-8 solution (Cat. No. 40203ES60, Yeasen, Shanghai, China) was added to each well in accordance with the instructions of the CCK-8 kit. After an incubation period of 1-2 hours at 37°C, the absorbance was quantified at a wavelength of 450 nm via a microplate reader (Varioskan LUX, Thermo Fisher Scientific).

### Transwell assay

After the Matrigel was liquefied at 4°C for 12 to 24 hours, the gel was diluted with medium at a ratio of 1:6. A 50 μl volume of diluted Matrigel (Cat. No. 3356234, BD, NJ, USA) was added to the upper chamber to facilitate coating the filter membrane, which was then placed in the incubator to dry (4 h). The concentrations of H460 and PC9 cells were adjusted to 1 × 10^6^ cells/ml. Next, a 100 μl cell suspension was deposited into the upper chamber. Subsequently, 600 μl of a solution containing 10% fetal bovine serum was added to the lower chamber. The cells were subjected to a 24-hour incubation in a 5% CO_2_ incubator maintained at 37°C. The chamber was subsequently removed and washed with PBS 3 times. The chambers were immersed in 95% ethanol for 5 minutes. Subsequently, the chambers were subjected to staining in a 0.5% crystal violet solution for 10 minutes, after which any unbound staining solution was carefully removed with PBS. The cells present on the upper surface of the filter membrane were delicately removed with a cotton swab, after which the cells on the lower side of the filter membrane were observed under a microscope.

### EdU staining

H460 and PC9 cells were seeded in 24-well plates at a density of 1 × 10^5^ cells/well. Following a 24-hour incubation, the cells were incubated with an EdU solution (Cat. No., ST067, Beyotime, Shanghai, China) for 2 hours. Subsequently, the cells were fixed using 4% paraformaldehyde for 60 minutes and washed twice with PBS. The nuclei were stained using 4′,6-diamidino-2-phenylindole (DAPI) staining solution (Cat. No., C1005, Beyotime, Shanghai, China) and flushed with PBS (3 times). Eventually, images were captured utilizing a fluorescence microscope from Olympus, Japan.

### Western blot

H460 and PC9 cells were collected and mixed with 350 μl of RPIA protein lysis solution (Cat. No., P0013B, Beyotime, Shanghai, China). Then, the samples were placed on ice for 15 min and centrifuged for 15 min at 4°C with a centrifugation radius of 8 cm and 12 000 r/min to obtain the supernatant. For the extraction of nuclear proteins, the Nuclear and Cytoplasmic Protein Extraction Kit from Beyotime (Cat. No., P0027 Shanghai, China) was used. After the concentration was adjusted, the proteins were boiled for 10 min to denature. SDS-PAGE gels were prepared for electrophoresis by dispensing 40 μg of each well. Following wet transfer at 300 mA for a duration of 120 minutes, the PVDF membrane (Cat. No. 36126ES03, Yeasen, Shanghai, China) was blocked (1 h, RT), followed by the addition of dilutions of HAMP antibody (Abcam, ab30760, 1:500), rabbit anti-GAPDH antibody (dilution, 1:2000, Cat. No. ab9485, Abcam, Cambridge, UK), anti-Lamin B receptor/LBR (dilution, 1:1000, Cat. No. ab32535, Abcam, Cambridge, UK), anti-nuclear factor kappa B (NF-κB) p65 antibody (dilution, 1:1000, Cat. No. ab32536, Abcam, Cambridge, UK), and anti-NF-κB p65 (phospho-S536) antibody (dilution, 1:1000, Cat. No. ab76302, 1:1000, Abcam, Cambridge, UK) at 4°C overnight. After three rounds of 10-minute washing with TBST, the sections were incubated with a goat anti-rabbit secondary antibody conjugated with horseradish peroxidase (1 h, RT). After another series of three 10-minute TBST washes, the protein profiles were evaluated via the Super ECL Detection Reagent ECL (Cat. No. 36208ES60, Yeasen, Shanghai, China). The resultant protein band images were subjected to analysis via software, and the optical density of GAPDH served as the internal reference for adjusting the optical density of the target protein.

### Establishment of NSCLC mouse xenograft models

A total of twenty-four BALB/c nude mice, aged between 6 and 7 weeks and weighing between 17.6 and 19.6 grams, were divided into groups for HAMP overexpression, HAMP downregulation, and their corresponding control groups, each comprising five mice. H460 cells were transfected, and a single-cell suspension at a concentration of approximately 1 × 10^7^ cells/ml was prepared and inoculated subcutaneously into the armpit of the left anterior upper limb of nude mice at 0.2 ml/mouse. The utmost diameter represented by “a” and the minimal diameter indicated by “b” of the transplanted tumor were gauged every 3 days subsequent to inoculation. The volume of the tumor was determined utilizing the formula V = ½ × a × b^2^. After 21 days, all the nude mice were allowed to acclimate through cervical dislocation. Subsequently, the tumors were removed, gauged, weighed, and fixed with formaldehyde to facilitate subsequent analysis. All animal-related protocols were granted approval by the Ethics Committee at the Fourth Hospital of Hebei Medical University.

### Immunohistochemistry

The tumor specimens derived from the nude mice were procured, treated with 4% PFA for fixation, and subsequently embedded in paraffin. After the paraffin sections (4 μm) were subjected to gradient dewaxing and hydration, a 3% H_2_O_2_ solution was added, and the mixture was incubated for 15 minutes to prevent endogenous peroxidase activity. The sections were immersed in citrate buffer and subjected to microwave-based antigen restoration. Subsequently, 5% normal goat serum was added for blocking, after which the mixture was incubated for 15 minutes (RT). The HAMP antibody (dilution, 1: 500, Cat. No. ab30760, Abcam, Cambridge, UK) or Ki67 antibody (1:250, Cat. No. 31078ES50, Yeasen, Shanghai, China) was suitably diluted at a ratio of 1:100 and incubated overnight at 4°C. Subsequently, a horseradish peroxidase-labelled rabbit secondary antibody (1:200) was added, and the samples were incubated for 30 minutes at 37°C. This was followed by the gradual addition of a horseradish enzyme-labelled Streptomyces leukolein solution for a 30-minute incubation at 37°C. After DAB color development, the sections were stained with hematoxylin (2 min, RT), dehydrated and mounted with neutral resin. The sections were then scrutinized using an upright microscope.

### Statistical methods

For statistical analysis, SPSS Statistics 22.0 software was used. All the outcomes are presented as the means ± SD. A *t* test was used to assess disparities in the results between two groups, while one-way ANOVA was used for comparisons involving three or more groups. A significance threshold of *P* < 0.05 was established for statistical significance.

### Data availability

The data are available from the corresponding author upon reasonable request.

## RESULTS

### HAMP was upregulated in NSCLC tissues and cells

**Figure 1:**
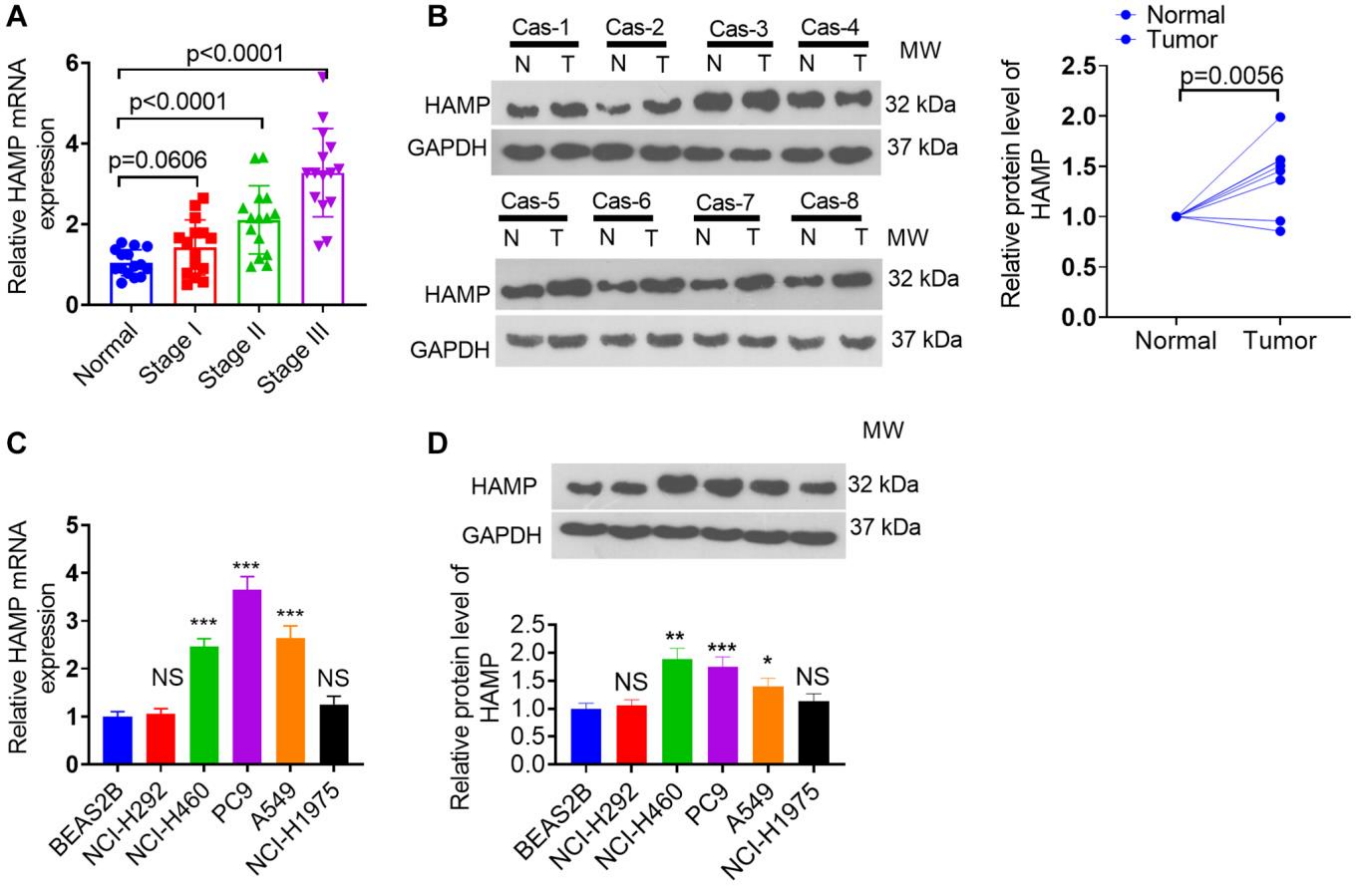
**HAMP expression changes in NSCLC tissues and cells.** (**A**, **B**) RT-PCR and western blot analyses were subsequently performed to assess HAMP mRNA expression in tumor tissues and adjacent normal tissues from individuals at distinct stages of NSCLC. (**C**, **D**) RT-PCR and western blot analyses of HAMP mRNA in NSCLC cells and normal cells. Abbreviation: NS: not significant. *P* > 0.05, ^*^P < 0.05, ^**^P < 0.01, ^***^P < 0.001 vs. BEAS-2B cells. *n* = 3.

RT-PCR was performed to determine the presence of HAMP mRNA across various stages in patients with NSCLC. HAMP mRNA was elevated in NSCLC patients compared to the normal control group. HAMP expression was further enhanced in patients with advanced-stage NSCLC (Figure 1A). The RT-PCR data were further validated by western blot analysis, which revealed an increase in HAMP protein levels in NSCLC tissues compared with neighboring normal tissues (Figure 1B). Additionally, the RT-PCR and western blot data revealed an increase in HAMP levels in PC9, H460, and A549 cells (Figure 1C, 1D). These findings imply that HAMP might augment cancer development in patients with NSCLC.

### Upregulation of HAMP promoted NSCLC cell proliferation and invasion

**Figure 2:**
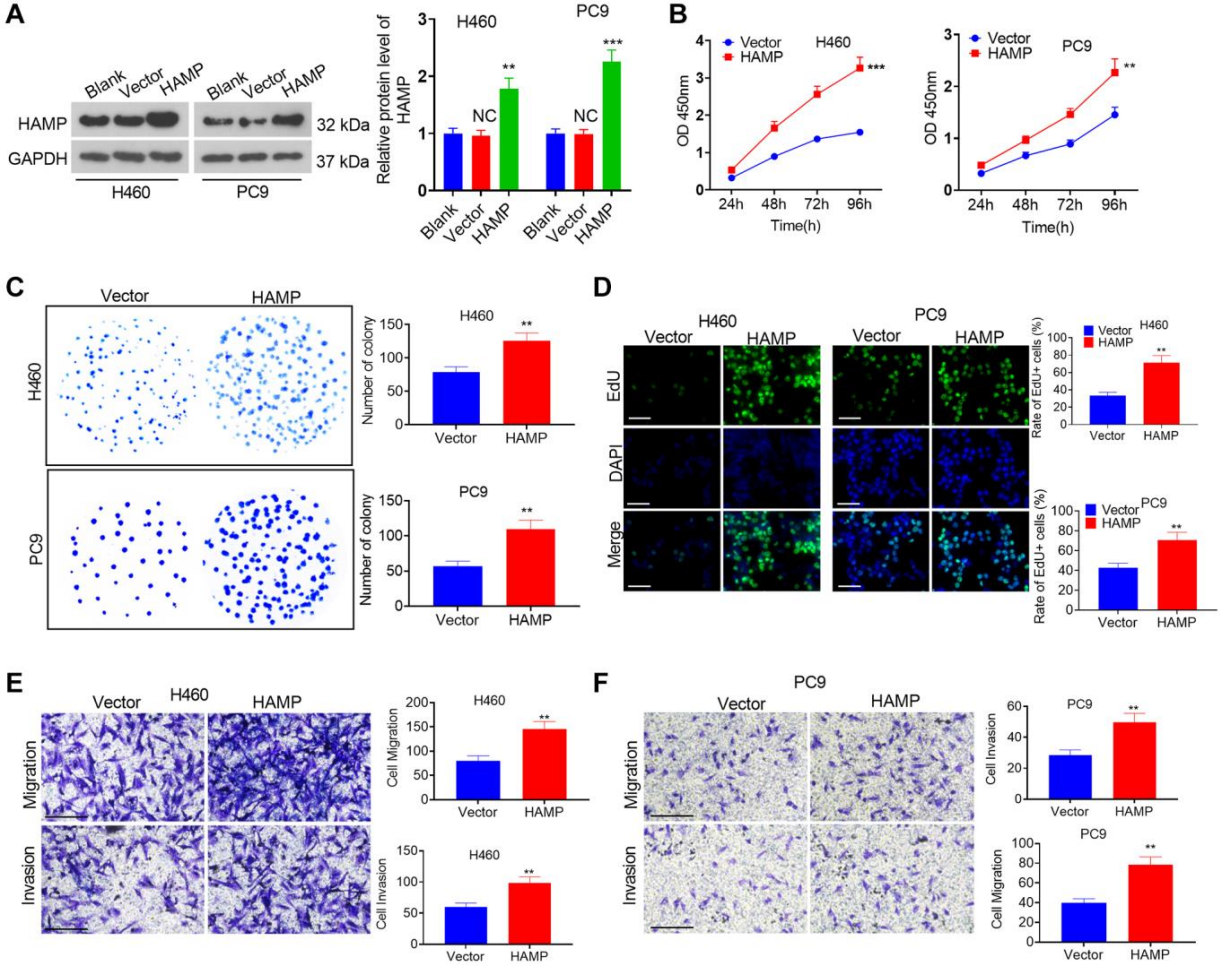
**The influence of HAMP upregulation on NSCLC cell proliferation and invasion.** (**A**) A cellular model for HAMP overexpression was developed in H460 and PC9 cells, and western blot analysis was carried out to evaluate HAMP protein levels. (**B**) Cell proliferation was assessed using a CCK-8 assay. (**C**) The ability of cells to form colonies was evaluated through a cell colony formation assay. (**D**) Cell proliferation was examined using an EdU staining assay. The scale bar is 50 μm. (**E**, **F**) Transwell assays were employed to evaluate the migratory and invasive properties of H460 and PC9 cells. Scale bar = 200 μm. *NS P* > 0.05 vs. *blank*, ^**^P < 0.01, ^***^P < 0.001 vs. vector. *n* = 3.

**Figure 3:**
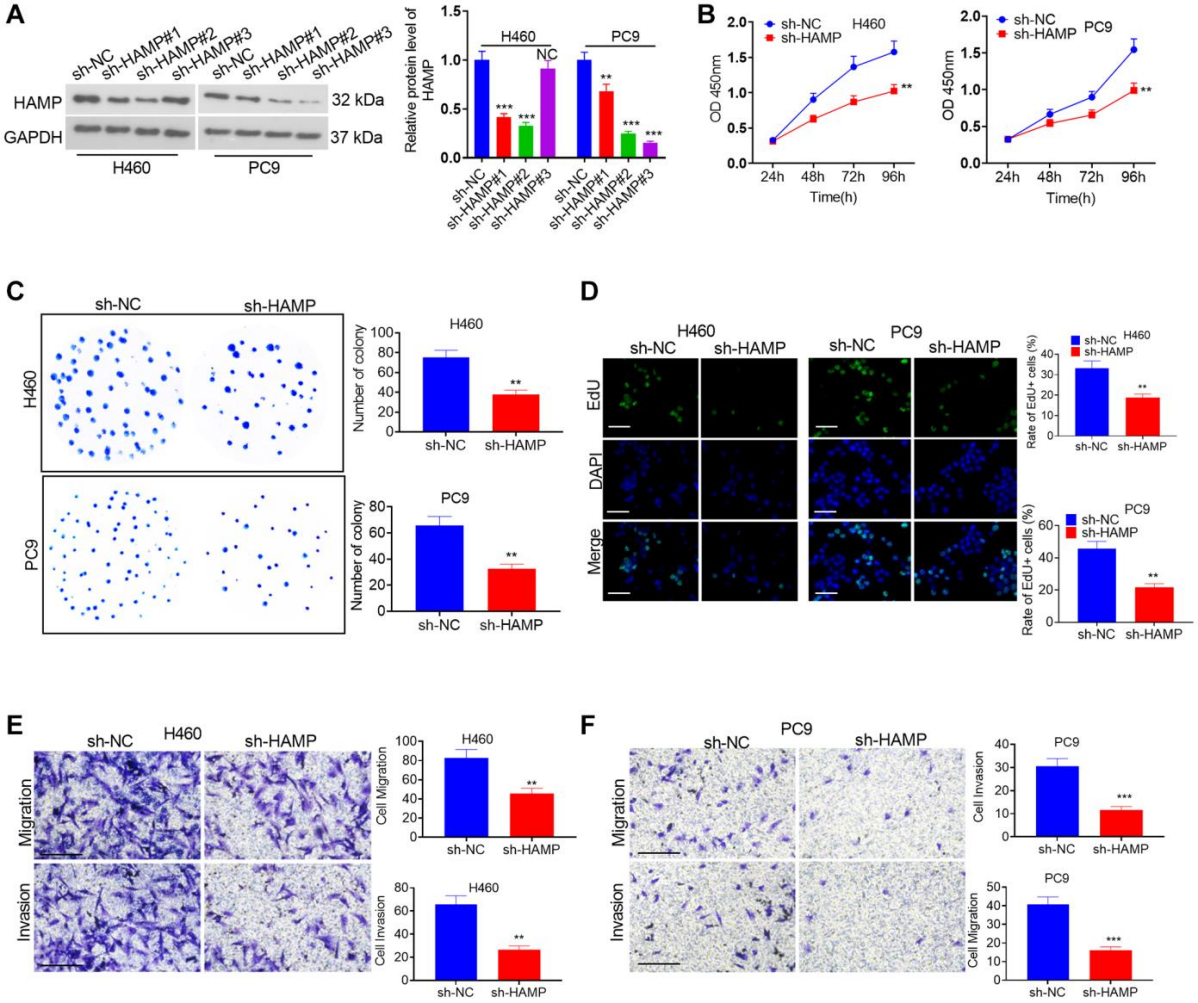
**The effects of HAMP knockdown on the proliferation and invasion of NSCLC cells.** (**A**) A cellular model for HAMP knockdown was established in H460 and PC9 cells, and western blot analysis was conducted to assess HAMP protein levels. (**B**) Cell proliferation was evaluated through a CCK-8 assay. (**C**) The ability of cells to form colonies was determined using a cell colony formation assay. (**D**) An EdU staining assay was used to evaluate cell proliferation. The scale bar is 50 μm. (**E**, **F**) Transwell assays revealed the migratory and invasive attributes of H460 and PC9 cells. Scale bar = 200 μm. *NS P* > 0.05, ^*^P < 0.05, ^**^P < 0.01, ^***^P < 0.001 vs. sh-NC (short hairpin RNA negative control). *n* = 3.

A cell model of HAMP overexpression was constructed in H460 and PC9 cells, which exhibited enhanced HAMP expression (Figure 2A). Next, functional assays were performed. The results of the CCK-8 and colony formation assays clearly demonstrated that overexpression of HAMP significantly enhanced the proliferation of NSCLC cells, specifically H460 and PC9 cells, compared with that in the vector cohort (Figure 2B, 2C). Moreover, the results of the EdU staining assay indicated that upregulated HAMP enhanced the percentage of EdU-positive cells (Figure 2D). Transwell assays revealed that upregulating HAMP conspicuously promoted the migratory and invasive capabilities of H460 and PC9 cells (Figure 2E, 2F). Furthermore, we performed a HAMP knockdown assay in these two cell lines. A HAMP knockdown cell model was successfully constructed (Figure 3A). Functional assays were subsequently performed. Subsequent to HAMP knockdown, the proliferation, invasion, and migration of H460 and PC9 cells were suppressed (Figure 3B–3F). Overall, HAMP aggravated the malignant potential of H460 and PC9 cells.

### HAMP promoted the growth of NSCLC cells *in vivo*

**Figure 4:**
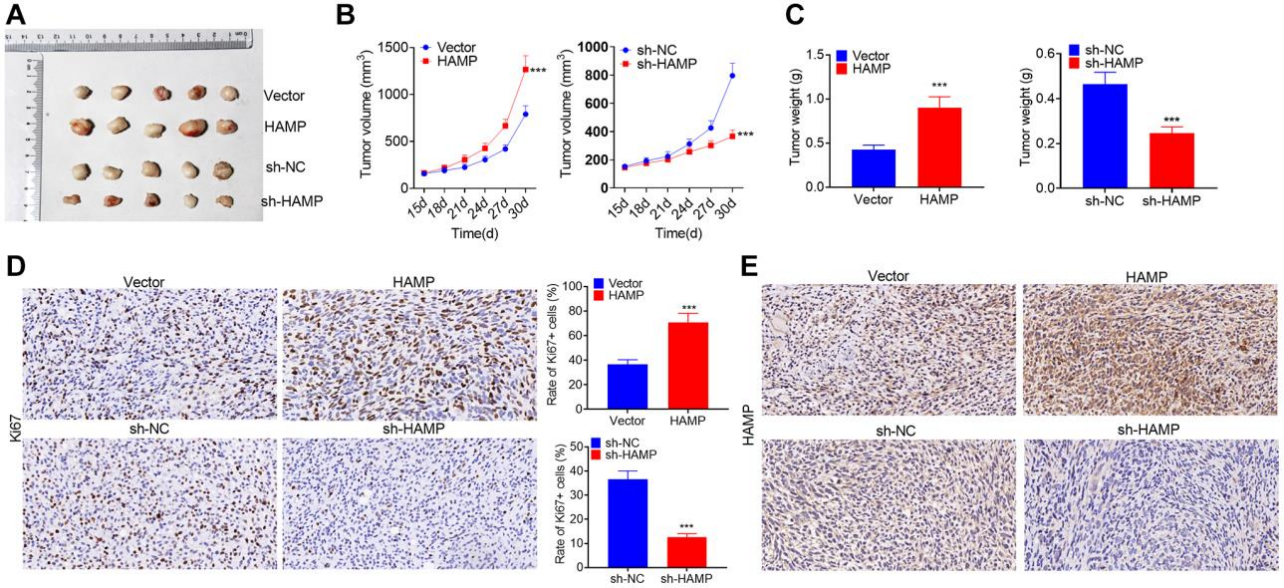
**HAMP promoted NSCLC cell growth *in vivo*.** HAMP-overexpressing and HAMP-knockdown cell models were used to construct NSCLC cell xenograft models. (**A**) Images of the tumors are shown. (**B**) Changes in tumor volume during the incubation days. (**C**) Tumor weights after the tumors were removed from the nude mice. (**D**) Ki67 staining was performed to evaluate cell proliferation. (**E**) IHC was used to detect HAMP in the tumor tissues. ^***^P < 0.001 vs. vector or sh-NC. *n* = 5.

*In vivo* assays were conducted utilizing nude mice. HAMP-overexpressing and HAMP-knockdown cell models were used to construct NSCLC cell xenograft models. The tumors grew faster after HAMP overexpression and grew more slowly when HAMP was knocked down (Figure 4A–4C). Ki67 staining was performed to assess cellular proliferation. The findings revealed an increased percentage of Ki67-positive cells within the HAMP-overexpressing cohort, while a reduction in Ki67-positive cells was observed in the HAMP-knockdown cohort (Figure 4D). Moreover, the IHC results confirmed that HAMP expression was increased in the HAMP-overexpressing cohort but was decreased in the HAMP-knockdown cohort (Figure 4E). Therefore, HAMP aggravated NSCLC cell growth *in vivo*.

### HAMP activated the NF-κB pathway in NSCLC cells

**Figure 5:**
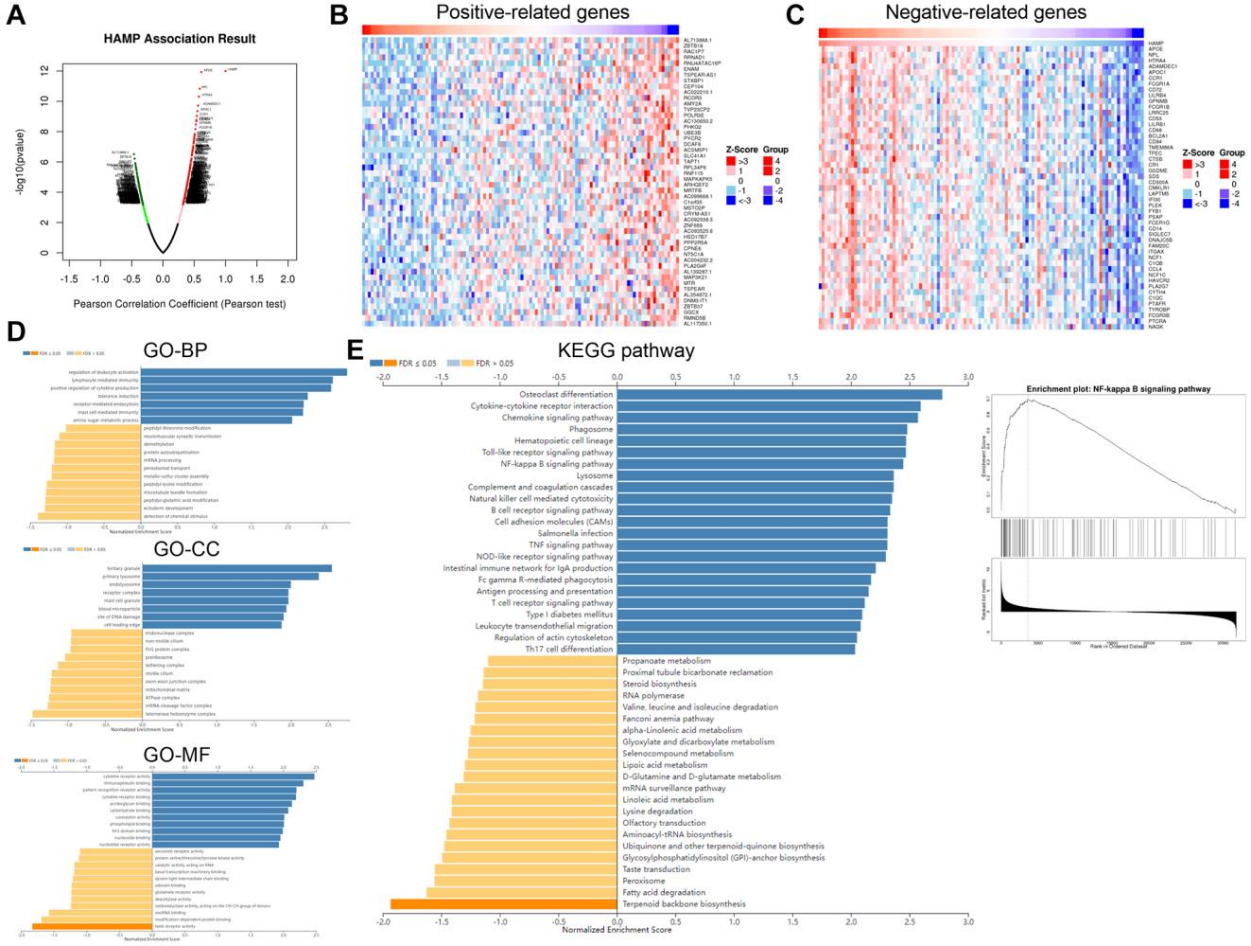
**HAMP might regulate the NF-κB pathway in NSCLC.** The genes that had significant correlations with HAMP in NSCLC were analysed through LinkedOmics. (**A**) The volcano map of those related genes is shown. (**B**, **C**) The heatmaps of those genes (positively related and negatively related genes) are shown. (**D**, **E**) Gene set enrichment analysis was conducted using the online tool from LinkedOmics. The analysis results showed that HAMP is associated with many Gene Ontology (GO) terms and enriched KEGG pathways.

**Figure 6:**
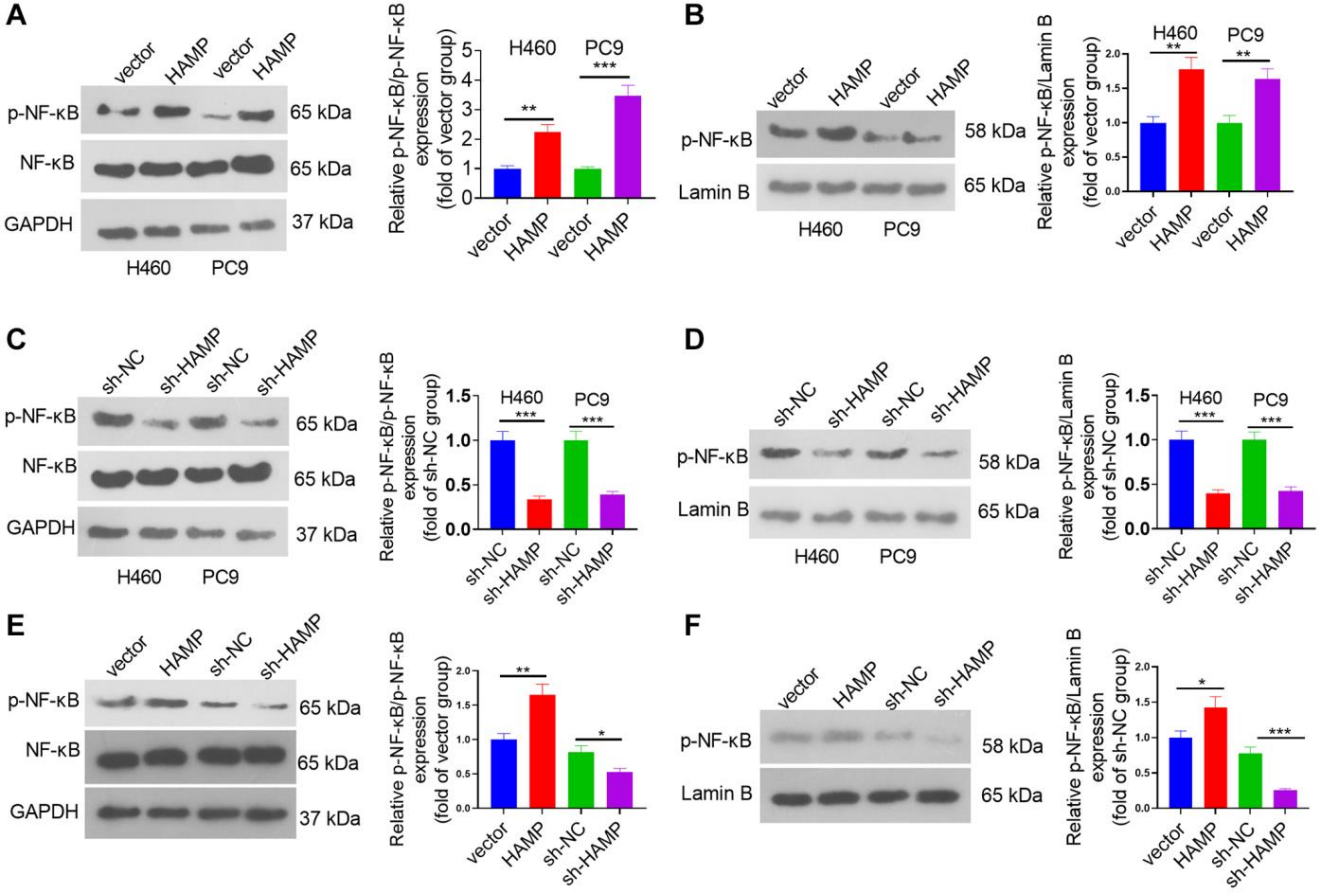
**HAMP upregulated the NF-κB pathway in NSCLC cells.** (**A**–**F**) HAMP-overexpressing or HAMP-knockdown H460 and PC9 cells were generated. Western blot analysis revealed p-NF-κB p65 protein levels in whole cells and in the nucleus. ^*^P < 0.05, ^**^P < 0.01, ^***^P < 0.001. *n* = 3.

To determine the downstream mechanism of HAMP in NSCLC, our initial step involved the analysis of genes exhibiting substantial correlations with HAMP in NSCLC by utilizing the LinkedOmics platform [23]. A volcano map of the related genes is shown in Figure 5A. The heatmaps of those genes (positively related and negatively related genes) are shown in Figure 5B, 5C. Next, gene set enrichment analysis was conducted using the online tool LinkedOmics. The analysis results showed that HAMP is associated with many Gene Ontology (GO) terms (Figure 5D). The enriched Kyoto Encyclopedia of Genes and Genomes (KEGG) pathways included the NF-κB signalling pathway, suggesting that HAMP potentially regulates this pathway in NSCLC (Figure 5E). We then performed western blotting to determine the protein levels of p-NF-κB p65 in whole cells and in the nucleus. HAMP overexpression enhanced p-NF-κB p65 in whole cells and in the nucleus, while HAMP knockdown had the opposite effect (Figure 6A–6F). Thus, HAMP might affect NSCLC progression by activating the NF-κB pathway.

### Inhibiting NF-κB pathway in NSCLC cells affected HAMP-mediated effects

**Figure 7:**
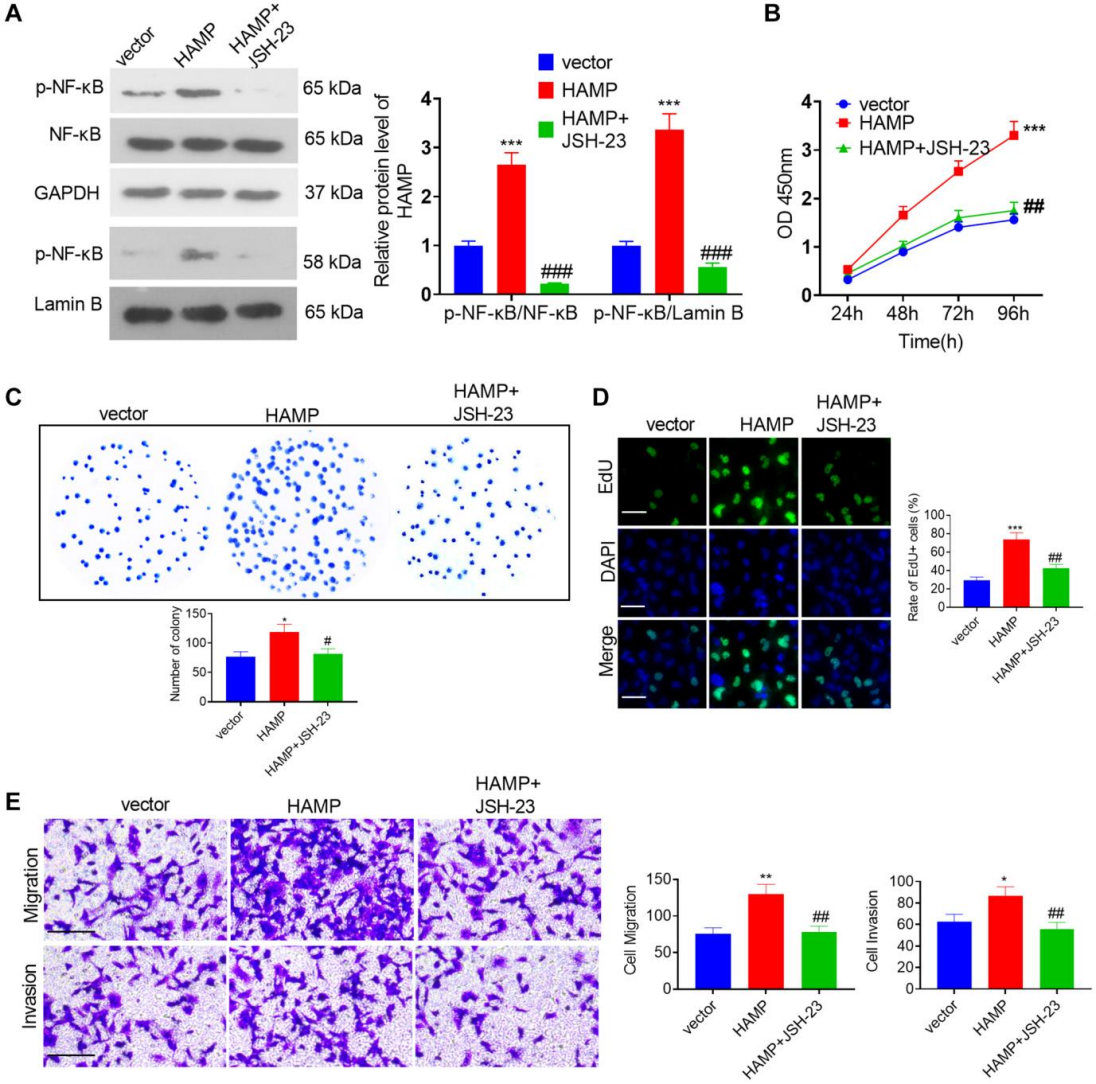
**HAMP upregulated the NF-κB pathway in NSCLC cells.** (**A**) JSH-23 (10 μM) was used for treating H460 with HAMP overexpression, and western blot analysis was carried out to evaluate p-NF-κB p65 protein levels in whole cells and in the nucleus. (**B**) Cell proliferation was assessed using a CCK-8 assay. (**C**) The ability of cells to form colonies was evaluated through a cell colony formation assay. (**D**) Cell proliferation was examined using an EdU staining assay. The scale bar is 50 μm. (**E**) Transwell assays were employed to evaluate the migratory and invasive properties of H460 cells. Scale bar = 200 μm. ^*^P < 0.05, ^**^P < 0.01, ^***^P < 0.001 vs. vector. ^#^*P* < 0.05, ^##^*P* < 0.01, ^###^*P* < 0.001 vs. HAMP. *n* = 3.

To confirm the role of NF-κB in HAMP-mediated effects, an NF-κB inhibitor was used. Western blot showed that JSH-23 inhibited the protein levels of p-NF-κB p65 in the whole cells and in the nucleus (Figure 7A). Then we performed functional assays. The data supported that compared with the HAMP overexpression group, JSH-23 can partly inhibit cell proliferation, migration and invasion of H460 cell (Figure 7B–7E). The result showed that JSH-23 significantly attenuated the malignant behaviors of NSCLC cells with HAMP overexpression.

### Regulatory mechanism of the lncRNA KCNQ1OT1/SNHG14/XIST-miR-873-5p-HAMP axis

**Figure 8:**
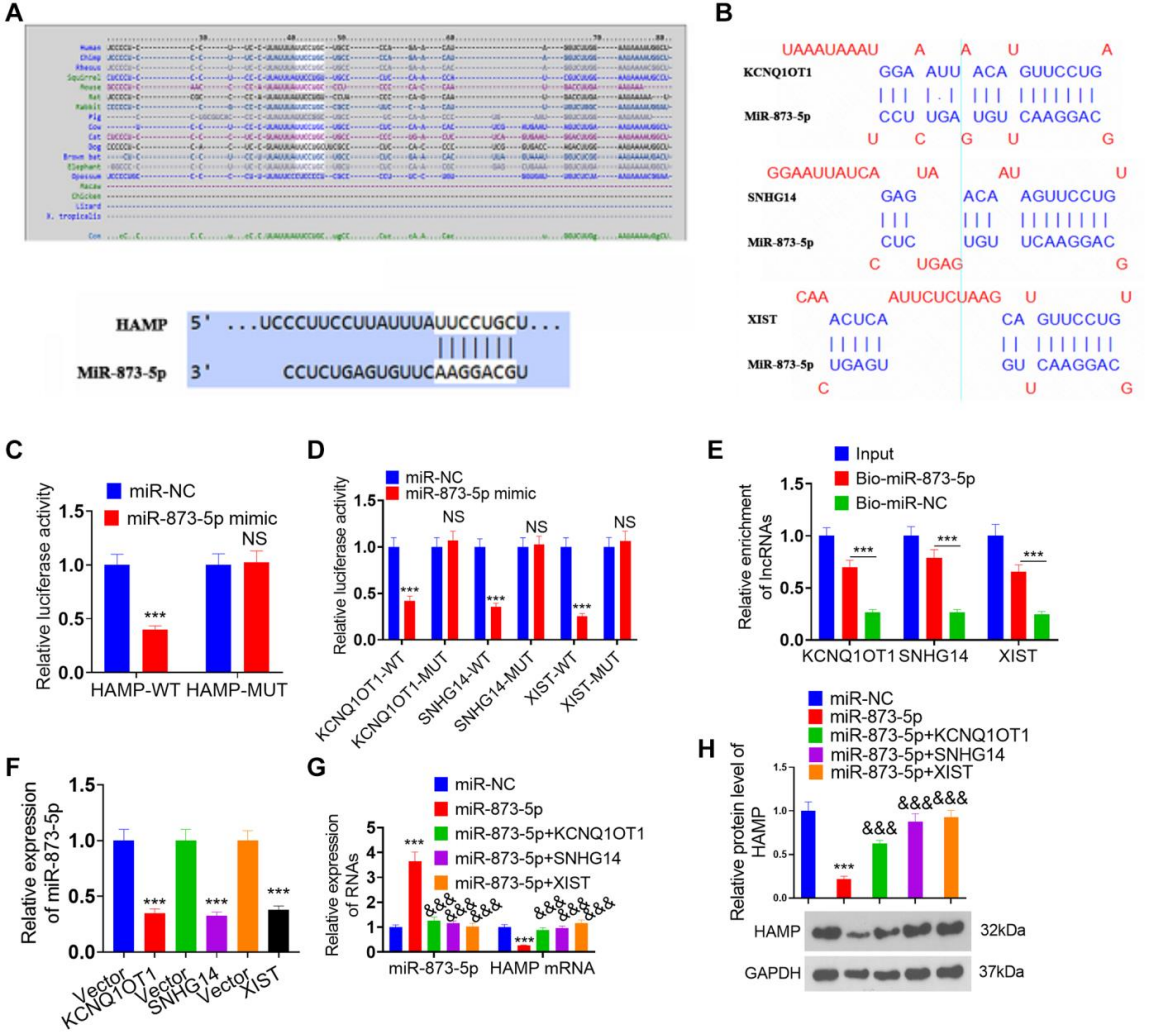
**Regulatory mechanism of the lncRNA KCNQ1OT1/SNHG14/XIST-miR-873-5p-HAMP axis.** (**A**) miR-873-5p targets the 3’UTR of HAMP mRNA, as determined via TargetScan. (**B**) The lncRNAs XIST, KCNQ1OT1, and SNHG14 potentially target miR-873-5p. (**C**, **D**) A dual-luciferase reporter assay revealed the interaction of miR-873-5p-HAMP, miR-873-5p-KCNQ1OT1, miR-873-5p-SNHG14, and miR-873-5p-XIST in 293T cells. (**E**) The interaction between miR-873-5p and KCNQ1OT1/SNHG14/XIST in 293T cell lysates was verified through pull-down analysis. (**F**) The expression levels of miR-873-5p were investigated following the transfection of KCNQ1OT1, SNHG14, and XIST overexpression plasmids into H460 cells. (**G**, **H**) H460 cells were transfected with HAMP overexpression plasmids, miR-873-5p mimics, or KCNQ1OT1, SNHG14, or XIST overexpression plasmids. Subsequently, HAMP mRNA and protein levels were evaluated. ^***^P < 0.001 vs. vector, ^&&&^*P* < 0.001 vs. miR-873-5p. *n* = 3.

Through bioinformatics analysis, we found that miR-873-5p targets the 3′UTR of HAMP mRNA (Figure 8A) and that the lncRNAs XIST, KCNQ1OT1, and SNHG14 potentially target miR-873-5p (Figure 8B). The dual-luciferase reporter assay revealed that the miR-873-5p mimic significantly enhanced the luciferase activity of 293T cells transfected with wild-type HAMP-WT, KCNQ1OT1-WT, SNHG14-WT, or XIST-WT, while the miR-873-5p mimic had no substantial effect on the luciferase activity of 293T cells transfected with wild-type HAMP-MUT, KCNQ1OT1- MUT, SNHG14- MUT, or XIST-MUT (Figure 8C, 8D). The pull-down results revealed that miR-873-5p decreased the KCNQ1OT1/SNHG14/XIST profile in A549 cell lysates (Figure 8E). After transfecting the KCNQ1OT1, SNHG14 and XIST overexpression plasmids into H460 cells, the miR-873-5p profile was significantly reduced, as determined by qRT-PCR (Figure 8F). The introduction of miR-873-5p mimics resulted in an increase in miR-873-5p levels in H460 cells, which led to the suppression of HAMP mRNA and protein expression. However, the effects of the miR-873-5p mimics were significantly reversed by transfecting the KCNQ1OT1, SNHG14 and XIST overexpression plasmids into H460 cells (Figure 8G, 8H). These findings revealed that miR-873-5p serves as a target miRNA of KCNQ1OT1/SNHG14/XIST, and in turn, HAMP is the direct target gene of miR-873-5p.

### Effects of the KCNQ1OT1/SNHG14/XIST-miR-873-5p-HAMP axis on H460 cell proliferation, invasion and migration

**Figure 9:**
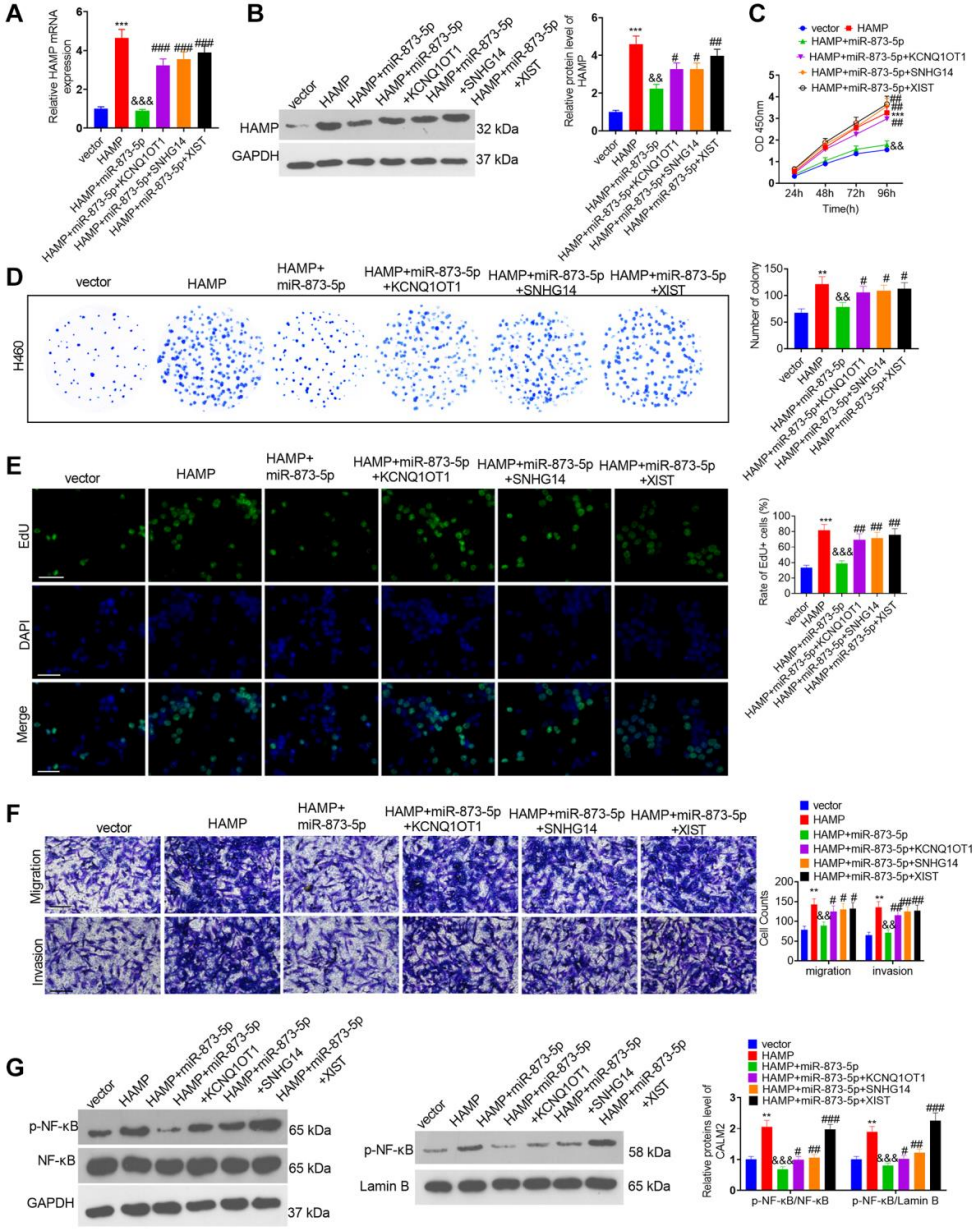
**The effects of the KCNQ1OT1/SNHG14/XIST-miR-873-5p-HAMP axis on H460 cell proliferation, invasion and migration.** The miR-873-5p mimics and the HAMP, KCNQ1OT1, SNHG14, and XIST overexpression plasmids were transfected into H460 cells. (**A**, **B**) The HAMP profile was evaluated using RT-PCR and western blot analyses. (**C**) Cell proliferation was assessed via a CCK-8 assay. (**D**) The ability of cells to form colonies was evaluated through a cell colony formation assay. (**E**) Cell proliferation was examined via an EdU staining assay. The scale bar is 50 μm. (**F**) Transwell assays revealed the migratory and invasive traits of H460 and PC9 cells. The scale bar is 200 μm. (**G**) p-NF-κB p65 protein levels in whole cells and in the nucleus were assessed through western blot analysis. ^**^P < 0.01, ^***^P < 0.001 vs. vector. ^&&^*P* < 0.01, ^&&&^*P* < 0.001, vs. HAMP; ^#^, ^##^, and ^###^ indicate *P* < 0.05, *P* < 0.01, and *P* < 0.001 vs. HAMP+miR-873-5p. *n* = 3.

The miR-873-5p mimics and the HAMP, KCNQ1OT1, SNHG14 and XIST overexpression plasmids were transfected into H460 cells. RT-PCR and western blotting confirmed that the miR-873-5p mimics attenuated HAMP expression, while KCNQ1OT1, SNHG14 and XIST mostly reversed this miR-873-5p-mediated effect (Figure 9A, 9B). The miR-873-5p mimics inhibited the proliferation of H460 cells overexpressing HAMP. KCNQ1OT1, SNHG14 and XIST overexpression enhanced cell proliferation (versus the HAMP+miR-873-5p cohort; see Figure 9C–9E). The migration and invasion of H460 cells were markedly reduced after miR-873-5p mimic transfection, and KCNQ1OT1, SNHG14 and XIST overexpression enhanced cell migration and invasion (vis-à-vis the HAMP+miR-873-5p cohort; Figure 9F). Moreover, western blot analysis revealed that the level of p-NFκB P65 within the whole cell and nucleus, which was reduced by miR-873-5p, was promoted after KCNQ1OT1, SNHG14 and XIST overexpression (Figure 9G).

## DISCUSSION

**Figure 10:**
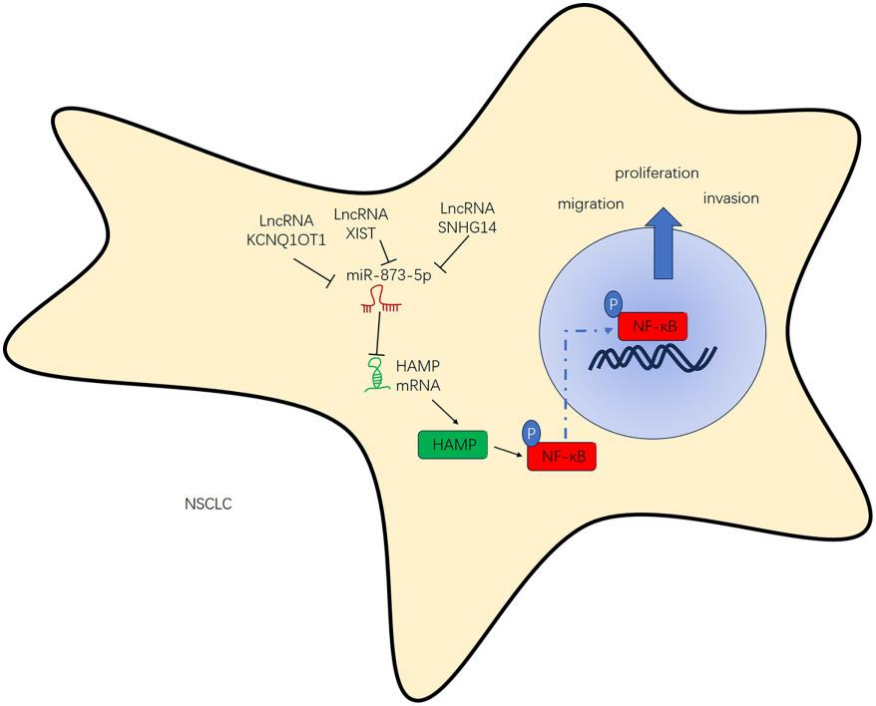
The regulatory mechanism of the KCNQ1OT1/SNHG14/XIST-miR-873-5p-HAMP axis in NSCLC.

Addressing these challenges requires continued efforts in research and drug development, exploring combination therapies, identifying novel targets, studying resistance mechanisms, improving diagnostic techniques, and enhancing access to personalized treatment options for NSCLC patients [24]. Presently, we investigated the expression, functions and mechanism of HAMP in NSCLC development. The results revealed that HAMP exerts oncogenic effects on NSCLC and that there is a positive regulatory relationship between KCNQ1OT1/SNHG14/XIST and HAMP (Figure 10).

Iron plays a pivotal role as an essential element for the survival of terrestrial life forms. Despite the rigorous regulation of iron metabolism within the body, disruption of the iron balance under specific conditions can result in variations in cell proliferation and modifications in both innate and adaptive immune responses [25]. In most cancer cells, cellular iron metabolism pathways are disrupted, which markedly affects tumor cell growth and proliferation. Furthermore, novel perspectives in cancer therapy emerge through therapeutic approaches centred on the targeting of altered iron metabolism-related proteins [26]. HAMP is a small peptide hormone that is primarily acknowledged for its function in iron metabolism [27] and may also play a role in cancer development and progression [28]. For example, human gastric cancer was found to have increased HAMP expression, which was correlated with increasing tumor stage. Furthermore, the binding affinities of the JAK/STAT3 signalling pathway and STAT3 for the HAMP gene promoter were substantially increased, indicating that there is a close connection between the modified expression of HAMP in human gastric cancer and the upregulation of IL-6-mediated JAK/STAT3 signalling [29]. Here, the findings revealed an increase in the HAMP profile within NSCLC cells and tissues. The HAMP overexpression group exhibited significantly elevated NSCLC cell proliferation, migration, and invasion, while HAMP knockdown had the opposite effect. Thus, HAMP has a tumor-promoting effect on NSCLC.

The NF-κB transcription factor family plays a vital role in inflammation and innate immunity and plays a pivotal role in numerous phases of cancer inception and advancement [30]. The NF-κB pathway plays indispensable roles in the progression of lung cancer and is a promising target for therapeutic intervention. For example, the adipokine angiopoietin-like protein 2 (ANGPTL2) can regulate tumor progression and metastasis by mediating lymphangiogenesis through activating the NF-κB pathway [31]. Antagonists that target NF-κB have been shown to be useful for clinical applications in lung cancer treatment [32]. In the liver, alcohol can activate TLR4-NF-κB signalling, while HAMP expression is suppressed after alcohol treatment, suggesting that TLR4-NF-κB signalling might participate in the suppression of hepcidin gene transcription in the liver [33]. However, after the administration of LPS or TNF-α, the mRNA level of hepcidin in peripheral blood leukocytes increases. Both anti-TNF-α antibody and NF-κB inhibition vigorously repress the levels of hepcidin mRNA elicited by LPS, indicating that the hepcidin mRNA profile in peripheral blood leukocytes elicited by LPS is contingent on NF-κB [34]. Here, we found that HAMP potentially regulates the NF-κB pathway. The western blot results also suggested that HAMP contributes to NF-κB pathway activation. The NF-κB inhibitor JSH-23 can partly reverse the enhanced cell proliferation, migration and invasion of H460 cells with HAMP overexpression. Therefore, we speculated that HAMP affects NSCLC progression by inducing NF-κB activation.

The molecular properties of lncRNAs and their key role in the complex network regulated by miRNAs, as well as the underlying mechanisms in normal and malignant cells, will help us better understand the potential of ceRNAs in cancer treatment [35]. KCNQ1OT1/SNHG14/XIST are three vital lncRNAs that have been found to regulate NSCLC progression [36–38]. In addition, miR-873-5p modulates NSCLC progression by regulating FOXM1 and β-catenin [39]. In this study, we revealed that miR-873-5p is the target miRNA of KCNQ1OT1/SNHG14/XIST and that HAMP is the target gene of miR-873-5p. KCNQ1OT1/SNHG14/XIST could upregulate HAMP mRNA and protein expression, and the introduction of miR-873-5p mimics reversed these changes by restoring HAMP mRNA and protein expression. *In vitro*, we also found that cotransfection of KCNQ1OT1/SNHG14/XIST and miR-873-5p mimics strongly affected lung cancer cell proliferation, invasion, and migration. These results suggest that KCNQ1OT1/SNHG14/XIST competitively combines with miR-873-5p to upregulate HAMP to form a complex regulatory axis involved in the pathological development of NSCLC.

To summarize, this research comprehensively explored the negative regulatory effect of KCNQ1OT1/SNHG14/XIST on miR-873-5p, which consequently augments the mRNA and protein levels of HAMP. This, in turn, amplifies the proliferation, migration, and invasion capacities of NSCLC cells by initiating the NF-κB pathway. The KCNQ1OT1/SNHG14/XIST-miR-873-5p HAMP axis could be implicated in the regulatory mechanism of NSCLC. Overall, this study provides a novel reference for the diagnosis, target therapy and molecular mechanism of NSCLC, which might help the treatment of NSCLC.
